# Buffalo

**Published:** 1896-08

**Authors:** 


### EDITORIAL.

### BUFFALO.

Our contemporary, the *Review*, in its July issue, exhibited great concern over the prospects of a poor programme at Buffalo. But there are very good reasons for its lack of information as to United States Association affairs. The senior editor's active direction of the *Review* for many years kept him in close touch with the Association and all its affairs. The junior editor has long been counted among the members of the Association, but his face is rarely seen at the meetings. His new duties have burdened his shoulders with new responsibilities, and we have no doubt he now feels that closer touch with Association work is essential to editorial management, and we shall have his counsel and assistance in many ways. The programme for Buffalo is loaded down with good things, and there are attractions for all. The time-limit will have to be enforced, and the five-minute rule in debate will be in order. The local arrangements are well in hand, and, under the direction of Chairman Hinkley, of the Committee of Arrangements of the U.S.V.M.A., there can be no failure to have everything in readiness for the reception of the members. The profession of Buffalo and vicinity are already organized and completing their plans, and the chief request of the officers of this body should be promptly complied with, and every veterinarian intending to be present should send the chairman of the committee word to that effect.

There should be no absentees among the Eastern members.

